# Improving the thermoelectric figure of merit

**DOI:** 10.1080/14686996.2021.1903816

**Published:** 2021-04-13

**Authors:** H. Julian Goldsmid

**Affiliations:** School of Physics, University of New South Wales, Sydney, Australia

**Keywords:** Thermoelectric energy convertor, thermoelectric materials, figure of merit, 50 Energy Materials, 210 Thermoelectronics / Thermal transport / insulators, 206 Energy conversion / transport / storage / recovery

## Abstract

The efficiency of a thermoelectric generator and the coefficient of performance (COP) of a thermoelectric heat pump are related to the hot and cold junction temperatures and a quantity known as the figure of merit, *zT*. During the second half of the twentieth century the figure of merit has gradually improved. This has come about through the selection of semiconducting materials with improved electronic properties and a small lattice thermal conductivity. Further advancements have been achieved by enhancing the scattering of phonons. There is also the possibility of improving the so-called power factor, that is the part of the figure of merit that contains the Seebeck coefficient and the electrical conductivity. However, it appears that it will be increasingly difficult to make further advances because of the manner in which these quantities vary with the Fermi energy. It is shown that this may set a practical limit on *zT*. Nevertheless, it may be possible to reach an efficiency or COP of about 40% of that of an ideal thermodynamic machine.

## Graphical abstract

**Figure:**
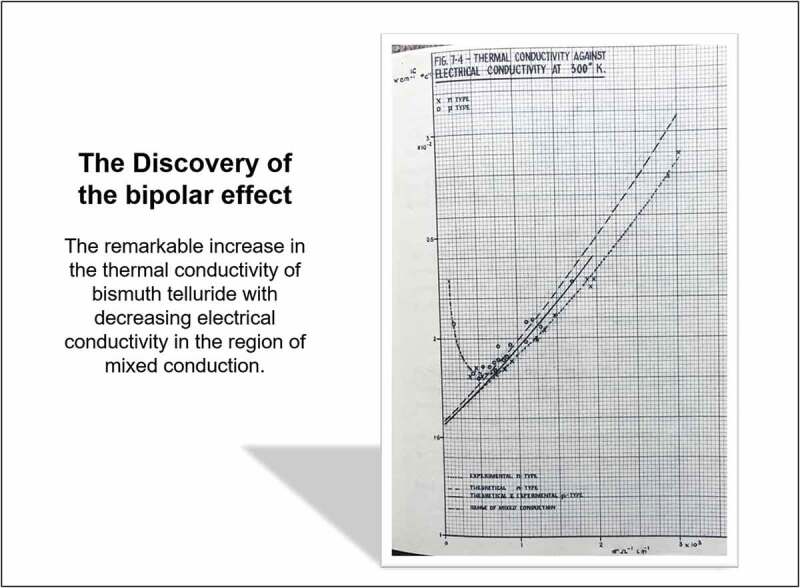

## 1. Introduction

The performance of a thermoelectric energy convertor, either as a heat pump or a generator, can be expressed in terms of the hot and cold junction temperatures and a quantity known as the figure of merit *ZT* [1].

(1) $$ZT=\frac{{\left(\alpha_{p}-\alpha_{n}\right)}^{2}T}{{\left\{{\left(\lambda_{p}/\sigma_{p}\right)}^{1/2}+{\left(\lambda_{n}/\sigma_{n}\right)}^{1/2}\right\}}^{2}}$$

*Here α* is the Seebeck coefficient, σ is the electrical conductivity and λ is the thermal conductivity. The subscripts p and n indicate the positive and negative branches respectively.

We suppose that the temperature difference, Δ*T*, between the junctions is much smaller than the absolute temperature, *T*. Then, if the device is used as a generator, the efficiency is

(2) $$\eta =\frac{\Delta T}{T}\frac{\left(M-1\right)}{\left(M+1\right)}$$

where *M* is equal to (1 + *ZT*)^1/2^. Likewise, the coefficient of performance in the heat pump mode is

(3) $$\phi =\frac{T}{\Delta T}\frac{\left(M-1\right)}{\left(M+1\right)}$$

under the same condition Δ*T* ≪ *T.*

It is common practice to make use of a figure of merit *zT* for a single material defined as α^2^σ*T*/*λ*. The figure of merit for a thermocouple is obviously related to the figures of merit *z*_p_ and *z*_n_ of the two branches and may well be close to the average of these two quantities. However, this may not always be the case. For example, if one of the branches is a superconductor its figure of merit will be zero and the average will be half the figure of merit of the other branch whereas the figure of merit of the couple will be equal to that of the active branch. In general, we need to know the ratio of the electrical conductivity to the thermal conductivity for each of the two branches before we can obtain the figure of merit of the couple. With this reservation we shall use the single-material figure of merit in our discussion here.

## 2. The optimum Seebeck coefficient

Nowadays effective thermoelectric materials are invariably semiconductors [2] in which the carrier concentration can be controlled by doping with acceptor or donor impurities. These impurities also determine the sign of the Seebeck coefficient. In general, we are interested in materials in which either electrons or positive holes are dominant. Minority carriers reduce the magnitude of the Seebeck coefficient and increase the thermal conductivity through bipolar conduction.

When carriers of only one sign are present, the magnitude of the Seebeck coefficient rises as the carrier concentration falls. If the energy gap is large enough it is possible for the Seebeck coefficient to rise to the order of ±1000 μV/K or more but the electrical conductivity is then exceedingly small. The product α*^2^σ*, known as the power factor, is highest when the Seebeck coefficient is of the order of ±200 μV/K. The Seebeck coefficient that yields the highest power factor is close to that which gives the highest figure of merit as is evident when we take into consideration the electronic contribution to the thermal conductivity.

The optimum Seebeck coefficient does not change very much from one material to another. On the other hand, the electrical conductivities can differ greatly. The carrier concentration for a given Seebeck coefficient depends on the effective mass or, strictly speaking, the density-of-states mass, m*. The electrical conductivity also depends on the mobility, *μ*, of the carriers, being largest when the inertial mass is small. What is needed is a large value for the product *μ* (m*/m)^3/2^. This is most likely to be found in a multi-valley semiconductor. Thus, the most commonly used thermoelectric material, p-type or n-type bismuth telluride, has 6 valleys. An *N*-valley semiconductor is equivalent to *N* conductors all sharing the same crystal lattice.

## 3. The lattice thermal conductivity

In most semiconductors the thermal conductivity is dominated by the phonon contribution, *λ*_L_. It is not surprising, therefore, that a great deal of effort has been devoted to the search for materials with a small lattice thermal conductivity. There is a general rule that the lattice thermal conductivity will be smallest when the mean atomic weight is largest. This rule was demonstrated experimentally by Ioffe [3] and supported by the theory of Keyes [4]. However, that is by no means the last word on the subject. Perhaps the most useful guide in searching for materials with a small lattice thermal conductivity is the observation of Slack [5] that certain substances can be regarded as crystalline from the viewpoint of electrical conduction but as glass-like when phonon conduction is under consideration. He called these materials PGECs or phonon glass electron crystals. He gave examples among the skutterudites which have large unit cells that can accommodate loosely bound atoms.

One of the best ways of reducing the lattice thermal conductivity is the formation of a solid solution [6]. For example, the compound lead telluride is a useful thermoelectric generator material. Its figure of merit can be further improved by alloying it with the isomorphous compounds lead selenide or tin telluride. The disturbance of the short range order is effective in scattering phonons but the maintenance of the long range order means that the carrier mobility is unaffected.

The use of a solid solution is particularly effective in silicon-germanium alloys. The lattice thermal conductivity of pure silicon is exceptionally large; its value of over 100 W/m K means that silicon is a better conductor of heat than many metals. However, the formation of a solid solution with germanium can reduce the lattice thermal conductivity to about 5 W/m K. It has, therefore, been possible to make useful thermoelectric generators from silicon-germanium alloys [7].

Another possible way of reducing the lattice thermal conductivity is by making the crystal size small. In most materials the phonons are predominantly scattered by other phonons. However, it has long been known that phonons can also be scattered at the crystal boundaries. This effect is usually most obvious at low temperatures when the mean free path of the phonons is greatest. At first sight it is not expected that boundary scattering of phonons would have a significant effect at ordinary temperatures. However, a substantial amount of heat is carried by low frequency phonons and these have a long free path and are more likely to be scattered on grain boundaries. The effect should be greatest for solid solutions because alloy scattering preferentially affects the high frequency phonons [8]. This means that much of the heat transport in solid solutions is carried by the low frequency phonons, the very group that is sensitive to boundary scattering.

By one means or another it has been possible to obtain materials with a lattice thermal conductivity close to that of glass, that is of the order of 0.25 W/m K. Further advances depend on an improvement of the product *μ* (m*/m)^3/2^.

## 4. The electronic properties

The value of μ (m*/m)^3/2^ for p-type bismuth telluride at room temperature is 560 cm^2^/V s. This is approached in few other materials, though it is equal to 2200 cm^2^/V s for electrons in pure silicon. [A table of semiconductor properties is to be found in the CRC Handbook of Chemistry and Physics, 91^st^ Edition, 2010–2011]. One might expect, then, that the power factor should be four times larger for silicon than for bismuth telluride. However, this turns out not to be the case. The electron mobility falls rapidly as impurities are added to silicon in order to optimize the carrier concentration. Ionized-impurity scattering is less noticeable in bismuth telluride because its much larger dielectric constant shields the electrons. It is just possible that a high electron mobility might be maintained in heavily-doped silicon if the necessary impurities are present as charged nanoparticles rather than as ions [9].

Hicks and Dresselhaus [10] actually proposed that nanostructured thermoelectrics would have an improved power factor compared with that of bulk material. This has prompted studies of nanostructured material and indeed a figure of merit *ZT* as large as 2.4 was reported for a nanostructured bismuth telluride alloy [11]. However, this large value has yet to be confirmed. In any case, even if the improvement is real, it is due to a reduction of the lattice thermal conductivity rather than any change in the electronic properties. Nevertheless, the adoption of nanostructures does offer the prospect of improving *zT* in the future.

It is now realized that the relationship between the Seebeck coefficient and the electrical conductivity in any given semiconductor is not unique. Thus, one of Ioffe’s suggestions was the deliberate introduction of ionized impurities to scatter preferentially the low energy carriers [1]. The reduction in mobility could be more than compensated by an increase in the Seebeck coefficient. This effect is likely to be of only marginal significance for most materials but it could be important when the energy gap is small or even negative as it is for bismuth. Bismuth would have a value of *zT* in excess of unity if conduction by positive carriers could be suppressed or if the low energy carriers could be preferentially scattered. It is quite possible that bismuth and bismuth-antimony will come into prominence as thermoelectric materials at ordinary temperatures through some such effect. A considerable enhancement of the figure of merit in bismuth has been reported for samples produced by a chemical exfoliation – spark plasma sintering process [12].

## 5. A barrier to further improvement

In selecting thermoelectric materials many workers use a dimensionless quantity *β* [13] that is given by

(4) $$\beta ={\left(\frac{k}{e}\right)}^{2}\frac{\sigma_{0}T}{\lambda_{L}}$$

*where k* is Boltzmann’s constant and *σ_0_* depends on the mobility and effective mass according to

(5) $$\;\sigma_{0}=2e\mu {\left(\frac{2\pi m^{*}kT}{h^{2}}\right)}^{3/2}$$

*β* has a value of 0.4 for a bismuth telluride alloy with *ZT* then equal to about 1. *β* would be equal to about 3.2 for a material with μ(m*/m)^3/2^ equal to its value for pure silicon combined with a glass-like lattice thermal conductivity. Such a material with an optimized carrier concentration should have *ZT* equal to about 4 and a Seebeck coefficient approaching ±400 μV/K. This would seem to be the best that one might hope for in the near future.

**Figure 1.**
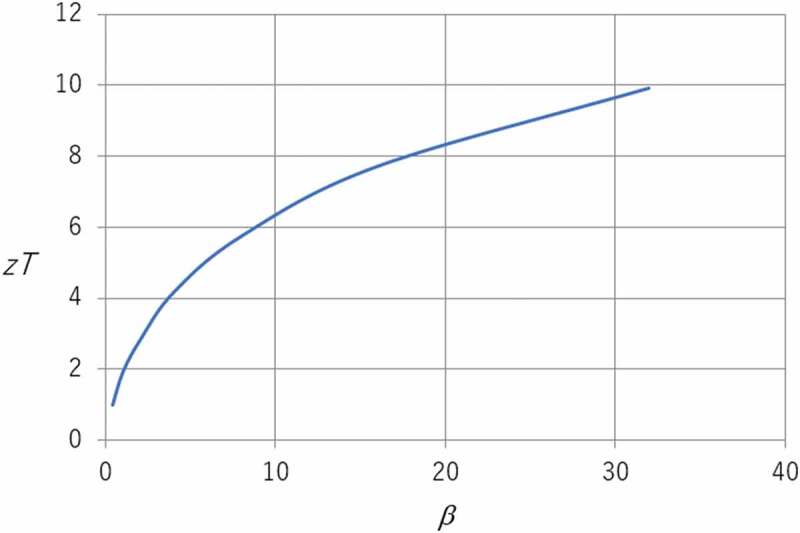
Plot of *zT* against the materials parameter β

In fact, even if larger values of β are forthcoming, it may still be difficult to obtain much greater values of *ZT*. The problem is that the Seebeck coefficient varies more or less linearly with the Fermi energy whereas the carrier concentration has a near exponential dependence. As β increases, the optimum Seebeck coefficient becomes somewhat larger but the carrier concentration becomes much smaller. Figure 1 shows how *zT* is expected to vary with β. Once *zT* becomes greater than unity, further improvement requires much larger values of β. β is equal to about 0.4 for a bismuth telluride alloy with *zT *= 1 and has to rise to about 3.2 for *zT* to reach 4. A further doubling of β only allows *zT* to climb to about 5.

At the present time there are a few materials with *zT* close to unity near room temperature. This allows us to make single-stage thermoelectric refrigerators that cool to about 90° below room temperature. A thermoelectric heat pump operating with a temperature difference between the source and sink of 30 degrees has a coefficient of performance of about 1. With *zT* = 4 the coefficient of performance would rise to about 3 for the same hot and cold junction temperatures. This would make thermoelectric heat pumps very attractive but even now they can be a viable alternative to conventional devices. One of the most convincing demonstrations of the virtues of thermoelectric air conditioning was a long-running trial on the French railways [14]. A 20 kW unit was installed in a train operating a regular service out of Paris. The unit worked perfectly with no failures over a period of ten years.

Perhaps the most important potential application is electrical generation from a low grade heat source. If *ZT* were equal to 4 the efficiency would be nearly 40% of that of an ideal machine. It is remarkable that this could be achieved with a system having no moving parts and capable of operating over a wide temperature range.

## 6. Conclusions

In summary, following the significant improvements in materials during the second half of the twentieth century it should now be possible to make thermoelectric generators and heat pumps that can compete with conventional devices. However, because of the manner in which the Seebeck coefficient and the electrical conductivity depend on the charge carrier concentration, it will be increasingly difficult to attain further improvements in the figure of merit *ZT*. Nevertheless, with this quantity rising to about 4, thermoelectric energy converters should still find widespread application.
